# Retzius‐sparing robot‐assisted radical prostatectomy in renal transplant recipients

**DOI:** 10.1111/bju.16737

**Published:** 2025-05-06

**Authors:** Jeffrey J. Leow, Santhosh Nagasubramanian, Zafer Tandogdu, Ashwin Sridhar, Prabhakar Rajan, Prasanna Sooriakumaran, Benjamin W. Lamb

**Affiliations:** ^1^ Department of Uro‐oncology University College London Hospital at Westmoreland Street, University College London Hospitals NHS Foundation Trust London UK; ^2^ Centre for Cancer Cell and Molecular Biology, Barts Cancer Institute Queen Mary University of London London UK; ^3^ Department of Urology, The Royal London Hospital Barts Health NHS Trust London UK; ^4^ Cleveland Clinic London London UK; ^5^ University of Oxford Oxford UK; ^6^ Department of Urology Christian Medical College Vellore Vellore Tamil Nadu India; ^7^ All‐India Institute of Medical Sciences Jodhpur Jodhpur India; ^8^ Department of Urology, National Healthcare Group Tan Tock Seng Hospital Singapore Singapore

**Abbreviations**
DVC: dorsal vascular complex
RARP: robot‐assisted radical prostatectomy
RS‐RARP: Retzius‐sparing robot‐assisted radical prostatectomy
VUA: vesico‐urethral anastomosis

Prostate cancer is the most prevalent non‐skin solid malignancy among male renal transplant recipients. Curative treatment options for localised prostate cancer include robot‐assisted radical prostatectomy (RARP) and radiotherapy. The largest multicentre study of transperitoneal RARP post‐renal transplant included 41 men treated across four European centres between 2009 and 2019 [1]. While the standard anterior approach is feasible, it poses risks of damaging the transplant kidney and the vesico‐ureteric anastomosis during anterior dissection and development of the space of Retzius.

Since Galfano et al. [2] published the first series of Retzius‐sparing RARP (RS‐RARP) involving 200 patients in 2013, the technique whereby the key anterior structures of the bladder such as the endopelvic fascia, dorsal vascular complex (DVC), arcus tendineus, levator ani muscle, pubo‐prostatic and pubo‐vesical ligaments are preserved has gained popularity [3, 4]. The Milan team reported early continence rates of 92% and low 1‐year biochemical recurrence rates. Over the next decade, RS‐RARP, despite its technical complexity, was increasingly adopted worldwide. A 2022 meta‐analysis comparing RS‐RARP with standard RARP, incorporating four randomised controlled trials and six prospective observational studies, found that RS‐RARP was associated with significantly better continence at 3 and 6 months [5].

The use of RS‐RARP can be viewed as a safer option for renal transplant recipients [6], as it avoids any dissection close to the transplant kidney, which is usually located in either the left or right iliac fossa. Here, we provide a step‐by‐step guide on how to perform an RS‐RARP in renal transplant recipients.

## Preoperative Preparation

The diagnostic evaluation for prostate cancer in renal transplant patients mirrors that conducted for the general male population. A biopsy‐confirmed prostate cancer diagnosis and a serum total PSA test within 3 months of surgery are essential. Cross‐sectional imaging, including prostate MRI, is crucial for local and systemic staging. MRI provides detailed information about prostate anatomy, volume, membranous urethral length, and tumour foci, aiding surgical planning and patient counselling. For instance, nerve‐sparing techniques might be omitted if high‐grade cancer or capsular bulging is evident on imaging. Additionally, a CT scan can delineate the relationship between the transplant kidney, bladder, and prostate, facilitating surgical safety.

A step‐by‐step video is provided (Video S1) to illustrate the procedure.

### Port Placement

**Fig. 1:**
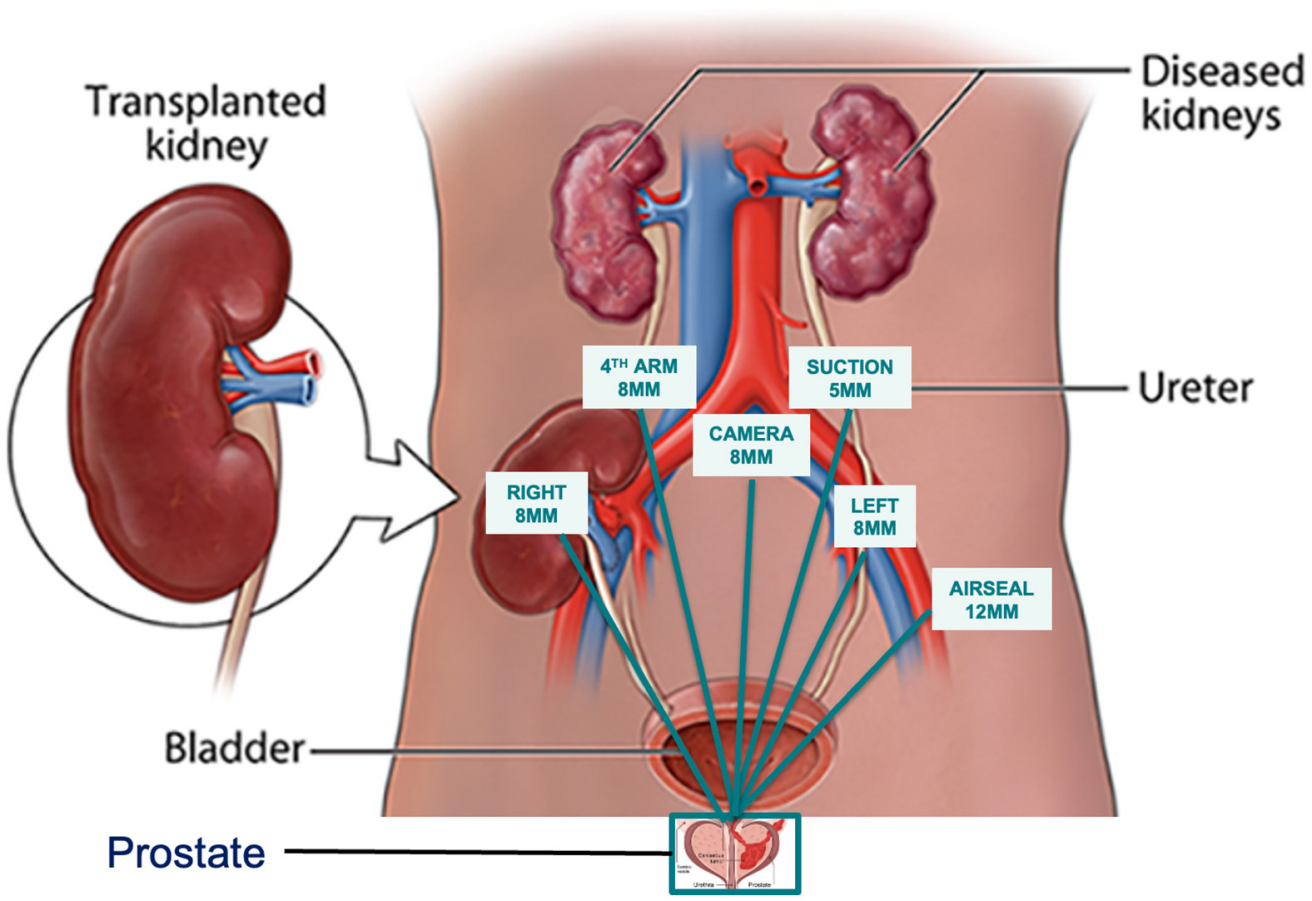
Port placement for Retzius‐sparing robot‐assisted radical prostatectomy in renal transplant patients.

In our practice, we start with a supra‐umbilical incision to gain intra‐abdominal access via Hasson's technique for camera port insertion. A transverse abdominis plane (TAP) block is administered under direct vision, injecting 0.375% bupivacaine to anaesthetise the anterior abdominal wall nerves (T6 to L1), reducing intra‐ and postoperative pain. To avoid injury to the transplanted kidney, the assistant is positioned on the contralateral side of the transplant. Two robotic ports ipsilateral to the transplanted kidney are placed; the right port is in a traditional position, and the fourth arm situated mid‐way between the camera port and the right arm, ~5 cm cranial, mirroring the 5‐mm assistant port (Fig. 1). Lastly, a 12‐mm assistant port is placed two finger‐breadths above the anterior superior iliac spine, on the contralateral side to the transplanted kidney. An AirSeal® device (Lawmed, ConMed) connected to the 12‐mm valveless assistant port maintains a more consistent intra‐abdominal pressure intra‐operatively. As increased intra‐abdominal pressure during robotic surgery can pose a risk to a transplanted kidney due to reduced renal perfusion, we typically set a pressure of 10 mmHg throughout most of the operation, and increase this transiently to 20 mmHg during the dissection through the DVC. Patients are placed in a steep Trendelenburg position.

## Posterior Dissection

The sigmoid colon is separated from any adhesions, then a peritoneal incision is made behind the bladder. Dissection continues in a plane inside the peritoneum, posterior to the vasa deferentia. The seminal vesicles are mobilised medially through blunt dissection in the avascular plane, avoiding electrocautery near the vesicle tips to protect the cavernosal nerves. During this initial phase of the operation, it is prudent to zoom out and visualise the transplanted kidney, to ensure that the robotic arms are not causing traction on or compression of the transplanted kidney. Posterior dissection proceeds with either an inter‐ or intra‐, or extra‐fascial technique, depending on the surgical plan [7].

## Prostatic Pedicles and Lateral Dissection

The prostatic pedicles are dissected, clipped with Hem‐o‐lok or metallic clips, and divided. Lateral dissection continues along the appropriate nerve‐sparing or extra‐fascial plane. A high or low release of the neurovascular bundle can be performed, depending on the position of the tumour, which is determined by preoperative biopsy and MRI findings. Higher releases improve nerve preservation, potentially benefiting postoperative continence and erectile function [8].

## Bladder Neck and Anterior Dissection

Attention turns next to defining the prostato‐vesical junction. With the Maryland bipolar forceps holding the bladder up anteriorly, the Prograsp forceps holds the pre‐dissected seminal vesicles to provide counter‐traction. In this manner, the detrusor slips and bladder neck fibres can be divided with a combination of cautery and blunt dissection, to preserve bladder tissue and delineate the urethra. A careful bladder neck‐sparing dissection may contribute to continence recovery. Bladder neck sparing may not be possible in patients with extensive disease at the base of prostate close to the bladder neck. After dividing the bladder neck and withdrawing the catheter, dissection continues over the anterior prostate surface until reaching the apex.

## Apex and Urethral Dissection

The DVC, located near the apex, is carefully separated from the prostate surface to preserve the striated urethral sphincter. Preserving the DVC may promote earlier continence recovery [8]. However, if anterior disease is confirmed by biopsy or imaging, anterior dissection proceeds through, or over the DVC, to ensure negative surgical margins. Careful apical dissection helps to maximise urethral length. The urethra is divided cold, avoiding thermal damage to the sphincter. The specimen is placed in an endobag. Haemostasis is ensured with cautery or absorbable sutures as needed.

## Vesico‐Urethral Anastomosis

The Prograsp forceps holds the bladder up in a relaxed position. The vesico‐urethral anastomosis (VUA) starts from the 12 o'clock position, outside in on the bladder side, with the full thickness of the bladder wall incorporated and exiting out of bladder mucosa. Sutures used include (i) absorbable barbed V‐loc 3/0 gold sutures with a 17‐mm 3/8 needle, (ii) absorbable barbed V‐loc 3/0 sutures on a 17‐mm 1/2 needle, or (iii) absorbable barbed Filbloc 3/0 sutures on a 5/8 needle. The urethral catheter is moved in and out during the fashioning of the VUA to allow the surgeon the visualise the urethra lumen and ensure the urethra mucosa has been incorporated. After the initial sutures are placed, the bladder is moved towards the urethra by bringing the Prograsp forceps caudally so that the sutures can be tightened without tension. Once the VUA is completed, a new 16‐Fr urethral catheter is inserted and a leak test is performed. Once no leak is confirmed, we instil 40 mL of 0.5% bupivacaine local anaesthesia to the bladder for 60 min, before free drainage is allowed postoperatively.

## Outcomes

We queried our prospectively maintained University College London Hospital (UCLH) prostatectomy database from January 2018 to July 2023 and identified 3507 patients who underwent non‐salvage RARP with curative intent. After a detailed chart review, we identified four patients who were kidney transplant recipients (Table 1).

**Table 1:** Characteristics of the renal transplant recipients (*N* = 4) who underwent non‐salvage robot‐assisted radical prostatectomy at University College London Hospital between January 2018 and July 2023 (*N* = 3507).

Characteristics		Patient 1	Patient 2	Patient 3	Patient 4
Age, mean (sd) years	68 (3.9)	63	70	67	72
Body mass index, mean (sd) kg/m^2^	25.5 (3.1)	23.24	23.8	30	25.1
**Baseline prostate cancer characteristics**					
Preoperative PSA, mean (sd) ng/mL	29.96 (27.6)	67.6	9.64	33.7	8.9
Preoperative Gleason score, *n*					
3 + 4	2		x	x	
4 + 3	2	x			x
Preoperative tumour stage, *n*					
T2	2	x	x		
T3a	2			x	x
**Postoperative oncological outcomes**					
Final pathology, *n*					
Gleason 3 + 4	2		x	x	
Gleason 4 + 3	2	x			x
Final tumour stage, *n*					
pT2c	2	x		x	
pT3b	2		x		x
Surgical margins, *n*					
Negative	3	x	x	x	
Positive >3 mm	1				x
Postoperative PSA, mean (sd) ng/mL	0.07 (0.11)	<0.01	0.195	<0.01	
**Postoperative outcomes**					
Length of stay, mean (sd) days	2.75 (1.5)	4	1	2	4
30‐day complications, *n*	0	Nil	Nil	Nil	Nil
Continence, number of pads/day		1	1	1	0
Time when continence was evaluated		8 months	12 months	6 months	5 months
**Renal function outcomes**					
Preoperative estimated GFR, mean (sd)	59.5 (17.8)	57	80	37	64
Postoperative estimated GFR, mean (sd)	58.3 (20.3)	72		35	68

All patients had intermediate‐ to high‐risk prostate cancer with Gleason Grade Group 2 or 3 disease. Most (three of the four patients) had negative surgical margins, and one patient had a positive surgical margin >3 mm. The location of the positive margin was noted to be intra‐prostatic (gland incision) at left anterior (4 mm). The mean postoperative PSA level was 0.07 (±0.11) ng/mL.

Patients stayed for a mean of 2.75 (±1.5) days; this was likely due to caution taken with postoperative monitoring of the kidney function. No patient experienced 30‐day complications or readmission.

There were no significant differences in preoperative vs. postoperative estimated GFR (59.5 ± 17.8 vs. 58.3 ± 20.3; *P* = 0.37) mL/min. Postoperative renal function was reliably obtained during the index hospitalisation for RARP.

Limitations of this study are that it included a small study cohort, and the lack of data on the lack of long‐term complications, and oncological and functional outcomes (urinary continence, erectile function, and transplant kidney function).

## Conclusion

We show that RS‐RARP is feasible and safe for renal transplant recipients, minimising risk of injury to transplant ureter and VUA, whilst offering an effective treatment option for localised prostate cancer.

## Disclosure of Interests

BWL has received funding from Cancer Alliances and NHS England for training MDTs in assessment and quality improvement methods in the UK; and honoraria for public speaking from Parsek. BWL received consultancy fees from Digital Surgery Ltd, MDOUTLOOK; and honoraria from Astra Zeneca and Astellas. AS is a proctor with intuitive surgical and receives payments for proctoring surgeons in retzius sparing RALP. ZT is an advisor for GSK in antibiotic development. PS receives research funding from Al‐Turki Holding and has worked as a paid consultant for Lindus Health, Smart About Health, and HeliosX over the past year. He has also received hospitality from Intuitive Surgical and Cambridge Medical Robotics in the past year. SN, PR and JL have disclosed no relevant interests.

## Supporting information


**Video S1.** Video of Retzius‐sparing robot‐assisted radical prostatectomy in renal transplant recipients: a step‐by‐step guide.
